# Effects of the Exclusive Enteral Nutrition on the Microbiota Profile of Patients with Crohn’s Disease: A Systematic Review

**DOI:** 10.3390/nu9080832

**Published:** 2017-08-04

**Authors:** Simona Gatti, Tiziana Galeazzi, Elisa Franceschini, Roberta Annibali, Veronica Albano, Anil Kumar Verma, Maria De Angelis, Maria Elena Lionetti, Carlo Catassi

**Affiliations:** 1 Department of Pediatrics, Università Politecnica delle Marche, 60123 Ancona, Italy; elisa.franceschini3@gmail.com (E.F.); robertannibali@hotmail.it (R.A.); vero.albano@ospedaliriuniti.marche.it (V.A.); mariaelenalionetti@gmail.com (M.E.L.); c.catassi@univpm.it (C.C.); 2 Laboratory of Metabolic Diseases, Università Politecnica delle Marche, 60123 Ancona, Italy; t.galeazzi@univpm.it (T.G.); anilkrvermaa@gmail.com (A.K.V.); 3 Department of Soil, Plant and Food Sciences, University of Bari A. Moro, 70126 Bari, Italy; maria.deangelis@uniba.it

**Keywords:** microbiota, metabolome, exclusive enteral nutrition, Crohn’s disease, IBD, next generation sequencing, 16S rRNA, *Faecalibacterium prausntzii*

## Abstract

The mechanisms behind the efficacy of exclusive enteral nutrition (EEN) in Crohn’s disease (CD) remain poorly understood, despite the high rate of treatment response. Evidence accumulated in the last 20 years suggests that a positive shift of the disrupted microbiota is one of the treatment effects. The purpose of this study was to critically review and summarize data reporting the microbiological effects of EEN in patients with CD. Fourteen studies were considered in the review, overall involving 216 CD patients on EEN. The studies were heterogeneous in methods of microbiota analysis and exclusion criteria. The most frequently reported effect of EEN was a reduction in microbiota diversity, reversible when patients returned to a normal diet. The effect of EEN on specific bacteria was very variable in the different studies, partially due to methodological limitations of the mentioned studies. The EEN seem to induce some metabolomic changes, which are different in long-term responder patients compared to patients that relapse earlier. Bacterial changes can be relevant to explaining the efficacy of EEN; however, microbiological data obtained from rigorously performed studies and derived from last generation techniques are largely inconsistent.

## 1. Introduction

Inflammatory Bowel Disease (IBD), including Crohn’s disease (CD), Ulcerative Colitis (UC) and Unclassified IBD (IBD-U), are chronic relapsing inflammatory disorders of the gastrointestinal tract resulting in significant morbidity. Despite the intense research on the underlying mechanisms, the etiology is still unclear. It has been proposed that IBD could be the consequence of an altered balance between the gastrointestinal (GI) microbiota and an inappropriate immune response to the GI bacterial community in subjects with a genetic predisposition [1,2]. The composition, the metabolic functions, and the roles of GI microbiota in IBD have been deeply investigated in the last 20 years, and the results have been recently reviewed and summarized [1,3,4,5]. Data suggest that bacterial dysbiosis is involved in pathogenesis, recurrence, and complications of IBD (especially increased abundance of Enterobacteriaceae, including adherent invasive *Escherichia coli* (AIEC) and a decreased abundance of Clostridiales, including *Faecalibacterium)* [5]. Additionally, changes in the microbiota composition (particularly at the mucosal level) potentially have prognostic and therapeutic implications [5]. At diagnosis, there is a window of opportunity to investigate the modifications of microbiota longitudinally, and studies conducted in untreated patients have shown interesting differences in children and adults with IBD [6].

Diet has a prominent role in IBD, and dietary factors have been implicated in the pathogenesis, recurrence, and treatment of IBD [7,8]. Exclusive enteral nutrition (EEN) is based on the administration of a liquid formula as the unique source of nutrition for a limited period of time (six to eight weeks). The EEN formulas differ in their protein and fat content and can be classified as elemental (monomeric), semi-elemental (oligomeric), or orpolymeric. Elemental formulas contain individual amino acids and glucose polymers and are generally low in fat. Semi-elemental formulas contain peptides of varying chain length, simple sugars, glucose polymers, or starch and fat, primarily as medium chain triglycerides (MCTs). Polymeric formulas contain intact proteins, complex carbohydrates, and mainly long-chain triglycerides (LCTs). A particular polymeric formula, enriched with an anti-inflammatory molecule (the transforming growth factor-beta 2, TGF-β2) has been designed specifically for patients with CD. European guidelines currently recommend EEN as the first-line therapy for inducing remission in pediatric luminal CD [9]. Such recommendations are based on a large amount of data showing rates of EEN-induced clinical remission up to 85% to 90%, and a rate of mucosal healing in 19% to 75% of patients [10,11,12]. The efficacy of EEN in CD is not correlated to the type of formula used (polymeric versus elemental) [13,14] or to the route of administration (oral versus naso-gastric tube) [15]. The rates of response to EEN are very high if compared to other treatment options in CD, while it is not useful in UC [16]. However, it is still unclear how this treatment works. Anti-inflammatory effects, avoiding specific dietetic components, restoration of the epithelial barrier, and alterations of the intestinal microbiota have been proposed and investigated as possible effects of EEN. The knowledge of these mechanisms can be important to understand the pathogenic events involved in IBD. Studies detailing changes in microbiota composition induced by EEN offer a unique chance to explore the isolated effect of a specific dietetic intervention in the IBD microbiota. Such studies require naïve treatment subjects (at diagnosis) and rigorous methodological criteria (i.e., exclusion subjects on antibiotics and probiotics and using new generation techniques of microbiota analysis).

Based on this background, we aimed to systematically review the existing literature on ‘the effects induced by EEN on intestinal microbiota’ in order to highlight the current knowledge on this topic, to reveal possible limitations of current studies, and to point out research gaps on this important issue.

## 2. The Normal Intestinal Microbiota: Techniques of Analysis

The human intestinal microbiota is represented by a complex system of bacteria, archaea, viruses, and fungi. In healthy subjects, the bacteria composing the intestinal microbiota primarily belong to two phyla that together represent 90% of the microbiota; the Firmicutes and the Bacteroidetes. Less represented phyla include Proteobacteria, Actinobacteria (including *Bifidobacterium*), Fusobacteria, Cyanobacteria, and Verrucomicrobia [17,18]. The characterization of the intestinal microbiota was originally based on microbiological techniques, with some limitations in the ability to culture a high proportion of intestinal bacteria [19]. More recently, culture efforts (culturomics) and new techniques in routine bacteriology have increased the number of identified species [20]. The introduction of molecular methods has further expanded the possibility of bacterial identification. More traditional methods, including electrophoresis and real-time quantitative polymerase chain reaction (PCR), are mainly based on the analysis of the 16S ribosomal RNA (rRNA) gene. This gene is ubiquitarious in all prokaryotes and contains both highly conserved and species–specific regions. The conserved regions work as amplification targets for PCR primers to extract one or more variable regions of the 16S rRNA gene. By using next-generation sequencing (NGS) technologies, the variable regions are replicated, producing thousands of sequenced fragments (reads), which are mapped to known 16S rRNA gene sequences of different microbial taxa such as genera or phyla or clustered according to their sequence similarity into “operational taxonomic units” (OTUs) [21]. The shotgun metagenomics sequencing approach has been developed in order to avoid some limitations of the more traditional techniques. Here, instead of targeting a specific genomic locus for amplification, all extracted DNA is broken-up into tiny fragments that are independently sequenced. These reads will be in part sampled from taxonomically informative genomic loci (e.g., 16S), while others will be sampled from coding sequences that provide insight into the biological functions encoded in the genome. As a result, metagenomic data provides the opportunity to simultaneously explore both the taxonomical and functional aspects of a microbial community [22].

## 3. Materials and Methods 

All studies (including cross-sectional, cohort, case-control, case reports, and series) evaluating the effect induced by the EEN or partial enteral nutrition (PEN) on microbiota composition or metabolomic profile in patients with CD, were considered eligible. Abstracts presented at meetings and articles not published in English were excluded. Studies conducted in children and adults were both considered. No publication date or publication status was imposed. Our search was applied to the Medline database using PubMed by combining key words for Crohn’s disease or Inflammatory Bowel Disease AND search terms for Enteral Nutrition, Exclusive Enteral Nutrition, Partial Enteral Nutrition, Exclusive Diet, or Polimeric Diet AND keywords for Microbiota, Microbiome, Metabolome, or Bacterial composition. All studies were published between 1950 (start of Medline) and January 2017. From each study, the following information were extracted: (1) Characteristics of the participants (number, age, disease’s status, method of diagnosis, concomitant therapies); (2) Type of microbiota analysis (biological sample and technique of analysis); (3) Nutritional treatment (type of formula, duration of treatment, exclusivity); and (4) Outcome of nutritional treatment and correlations with microbiota changes.

## 4. Results

### 4.1. Studies Characteristics

Fourteen studies were identified (one case-control report and 13 prospective studies, 12 of them including a control group) [23,24,25,26,27,28,29,30,31,32,33,34,35,36]. All the studies were considered relevant for the purpose of this review. The main characteristics and outcomes of the included studies are presented in Table 1.

Two studies were conducted in adults [25,26] and 12 in children [23,24,27,28,29,30,31,32,33,34,35,36]. Overall 462 subjects were enrolled, 314 of them having CD. Out the 216 enrolled CD subjects that received EEN, longitudinal microbiological data (including at least two consecutive fecal or biopsy samples during an EEN course) were available only for 150 patients. All the studies included subjects with active CD (at diagnosis or during a flare-up). In five studies, the use of antibiotics (in a period of time variable between one week and three months prior to enrollment) was considered an exclusion criteria [23,24,29,30,32]; one study excluded only patients on antibiotic or probiotic treatment during the study period [26]; and another study excluded subjects who had received probiotics in the two weeks before the start of the study [31]. The remaining eight studies did not indicate the use of antibiotics or probiotics as an exclusion criterion.

### 4.2. Methodologies of Microbiota Determination

The earliest study was conducted separating PCR-amplified fragments of 16S rDNA in polyacrylamide gels containing a gradient of denaturating agents (DGGE) or in a temperature gradient (TGGE) [23]. With this method, heteroduplexes of different amplicons (with different G/C contents) were dissociated at different positions in the denaturating or temperature gradient, resulting in a hold of migration. The result was a pattern of bands, which is characteristic of the bacterial community present in the sample. A combination of electrophoresis and real time PCR was used in the studies by Leach et al. and Gerasimidis et al., where bacterial 16S rRNA genes were amplified from stools using primers for different groups of bacteria, allowing specific analysis of each bacteria group [24,29]. Real time quantitative PCR was used in two further studies [25,29]. The most recent studies were performed using next generation sequencing (NGS) techniques [28,30,31,32,33,34,35]. Microbiota was extracted from fecal samples in all but one single case report, where ileal histological specimens were analyzed instead [28].

### 4.3. Overall Effects Induced by EEN on Microbial Composition, Diversity and Abundance

A significant decrease in the bacterial diversity during EEN treatment was observed in different studies using different techniques. Gerasimidis et al. demonstrated a decrease in the bacterial diversity richness (calculated as the total number of bands on the TTGE images) after 30 days of EEN (*p* = 0.041), persisting at the end of EEN (*p* = 0.037) [29]. In the study by Quince et al. diversity in species (represented by the Shannon diversity index) decreased already after two weeks of EEN (*p* = 0.037) [30]. In the same study, at the species level, EEN increased the microbiological distance between CD patients and healthy controls. Lewis et al. reported the same effect from increasing the distance from the healthy subjects microbiota composition with EEN [31]. A reduction in the number of operational taxonomic units (OTUs) was reported also by Kaakoush and collaborators [32]. Quince et al. quantified such reduction, demonstrating a drop of 0.6 points in the Shannon diversity index calculated at the genus level every 10 days of EEN [30]. This corresponds approximately to a reduction in genus diversity of 20% after one month of EEN. Conversely, in the small study by Schwerd et al., the Shannon diversity index was not affected by the nutritional treatment [33]. The authors attributed this unexpected result to the very stringent parameters used to filter OTUs. Also in the study by Shiga et al., species diversity was not reduced by EEN, in contrast with the effect induced by total parenteral nutrition (TPN) [26]. The reported reduction in microbial diversity seemed to correlate well with the achievement of clinical remission induced by EEN [32] and to reverse when patients returned to a normal diet [29,30,32].

### 4.4. Effects on Specific Bacterial Species or Strains

The effect of the EEN on specific bacteria species varied largely between the different studies. Quince et al. described a statistically significant reduction in 33 genera over the EEN course (including genera that were already less abundant in CD at baseline compared to controls, including *Faecalibacteria*, *Bifidobacteria*, and *Ruminococcaceae*) [30]. In the same report, only *Lactococcus* increased significantly with EEN (*p* = 0.017). A diet-induced reduction in the abundance of *Faecalibacterium prausnitzii* (belonging to Firmicutes phylum), a bacterium previously considered protective towards the development and flare-up of CD [37,38], was reported both by Jia et al. in adults (a significant decrease was observed for the A2-165 subgroup, *p* = 0.0046) and by Gerasimidis et al. (*p* = 0.023) and Quince et al. (decrease of −0.0144 log genera abundance, *p* = 0.068) in CD children [25,29,30]. However, in the paper by Kaakoush et al., the authors observed that different OTUs classified under *Faecalibacterium* responded differently to EEN therapy [32], and, in contrast, Schwerd et al. described an increase in the relative abundance of Firmicutes (*p =* 0.0227), particularly in members of the family Christensenellaceae, with no mention of changes in *F. prausnitzii* [33]. Among the Bacteroidetes, a significant reduction in the relative sequence abundance of the phylum (*p =* 0.039) was reported by Schwerd et al. [33], and a decrease in *Bacteroides fragilis* (*p =* 0.034) was described by Shiga et al. [26]. A trend for decreasing concentrations of *Bifidobacterium* genus, belonging to Actinobacteria phylum (at 60 days of EEN *p* = 0.120), was reported by Gerasimidis et al., with a subsequent increase when patients were back to a normal free diet (*p =* 0.031) [29]. In the recent report by Guinet-Charpentier et al., a decrease in genera from the Proteobacteria phylum (particularly *Sutterella, p* < 0.05) was observed, whereas *Alistipes* (*p* < 0.05) and *Bifidobacterium* (*p* < 0.1) increased over the course of EEN [34]. Table 2 summarizes specific bacterial changes induced by EEN.

### 4.5. EEN Induced Microbiota Changes and Relation to Disease Activity and Remission

The dietary treatment, in line with the literature, led to a high remission rate in CD subjects (up to 90%). The decline in specific bacteria, particularly the levels of *F. prausnitzii* [25,29] and *Bacteroides*-*Prevotella* [29], was found to correlate with the achievement of clinical remission. In the recent study by Guinet-Charpentier et al., patients responding to EEN and in clinical remission showed a reduction in *Dialister*, *Blautia*, unclassified Ruminococcaceae, and *Coprococcus* compared with patients in remission with other treatments such as anti-TNF and partial enteral nutrition (the clustering based on microbial distribution was significant with Monte Carlo, *p* value *=* 0.029, based on 10,000 replicates) [34].

In the recent study by Dunn and collaborators, interesting differences emerged between patients that achieved and maintained remission at week 24 after EEN (sustained remission-SR) and patients who did not achieve or maintain remission (non-SR) [35]. Species richness (estimated according to the Chao-1 index) was higher among the SR group (no statistical significance was shown in the text). Over the EEN course, species diversity tended to decrease in the SR group and to increase in the non-SR subjects. Furthermore, the taxonomic composition of the SR group was much more similar to healthy controls at the principle coordinate analysis than the non-SR group (no statistical calculation was made). *Akkermansia muciniphila* and *Bacteroides* were particularly prevalent in the SR group, whereas the non-SR group observed a prevalence of Proteobacteria. The authors reported that their ‘proposed microbiological model’ (based on the microbiota composition of pre-EEN samples) can predict sustained response to EEN with an accuracy of 80% [35]. The same group also looked at differences in the abundance of specific metabolic pathways in SR patients, compared to controls and non-SR patients. The authors initially identified eight metabolic pathways that differed significantly between CD patients and controls (*p* value < 0.1); the comparison of these eight pathways showed that SR patients were much more similar to controls compared to non-SR patients in the abundance of these pathways (non-SR patients differed significantly in the abundance of seven of the eight pathways, while SR-patients differed significantly from the controls in the abundance of two pathways only, *p* value < 0.05, after correction according to the method of Benjamini and Hochberg). However no significant difference was detected between SR and non-SR samples, possibly due to the small number of samples (10 in total) [36].

Finally, Kaakoush et al. reported re-colonization with specific microbial taxa belonging to six Firmicutes families and that increases in OTUs were correlated with disease recurrence) [32].

### 4.6. Effects on Metabolic Pathways

Short chain fatty acids (SCFAs) are produced in the intestinal lumen by the anaerobic fermentation of non-digestible dietary residues and endogenous epithelial-derived mucus. They are readily absorbed and used as energy sources by colonocytes and also by other tissues [39]. The fecal pool of SCFAs is correlated both to diet and to the microbiota composition and abundance. The three major luminal SCFAs are acetate (acetic acid), propionate (propionic acid), and butyrate (butyric acid). Butyrate is the most extensively studied, and its inflammatory properties are well documented [40]. The anti-inflammatory capacity of acetate is less documented, and interestingly it is also commonly used to induce inflammatory colitis in animal models [41,42], also suggesting pro-inflammatory properties.

Two studies specifically investigated the effects of EEN on the bacterial metabolism. Tjielstrom et al. [27] showed that EEN induced a significant reduction (*p* < 0.05) in the fecal median concentration of the acetic-acid. A parallel increase in anti-inflammatory SCFAs (butyric and valeric acids) was described in the same paper [27], although this change did not reach statistical significance. The same effects were not encountered in children with perianal CD. In the study by Gerasimidis et al., fecal pH and total sulfide increased, while butyric acid decreased during EEN, and these changes reverted to baseline on a free diet [29]. The metagenomics approach used by Quince et al. revealed that the effect of the EEN was associated with a reduction in the expression of genes involved in biotin and thiamine biosynthesis and a parallel increased expression of genes involved in spermidine/putrescine biosynthesis or in the shikimate pathway [30]. Changes in genes involved in the byosinthesis of vitamin B complex can potentially reflect a decrease in bacteria that express genes encoding these vitamins (e.g., bifidobacteria, *Lactobacillus reuteri*, and *E. coli*) or in the production of short or medium chain fatty acids that require these vitamins, or, alternatively, the supplementation of vitamin B complex by EEN may reduce bacterial production. The shikimate pathway is an alternative metabolic route for the biosynthesis of aromatic amino acids (phenylalanine, tyrosine, and tryptophan) employed by microorganisms and plants but not by animals and humans. The biosynthesis and transport of spermidine/putrescine and the shikimate pathway, implicated in the essential aminoacids synthesis, are both fundamental for cell growth and the overexpression of these genes can potentially indicate tissue regeneration [43].

In the recent study by Dunn et al., eight metabolic pathways were initially identified as substantially different in CD patients compared to controls, including pathways involved in xenobiotic and environmental pollutant degradation, succinate metabolism, bacterial HtpG, fatty acid metabolism, and the nucleotide-binding oligomerization domain (NOD)-like receptor signaling pathway. The abundance of pathways implicated in the degradation of environmental pollutants and xenobiotic degradation showed an increase in non-SR patients, whereas pathways involved in NOD-like receptor signaling were reduced in non-SR patients [36].

## 5. Discussion

The modification of the intestinal microbiota, commonly reported both in GI and non-GI diseases and widely described in IBD, is one principle potential mechanism behind the efficacy of EEN. In the last 15 years, some studies have tried to elucidate this aspect (which may further contribute to a general comprehension of IBD pathogenesis). Our review aimed to describe the literature on this specific topic, revealing a low number of underpowered and methodologically heterogeneous studies. However, the most recent trials, performed following strict inclusion criteria and using new generation techniques, led to intriguing results.

The origin of the microbial sample is a critical issue in studies describing intestinal bacterial composition. In fact, fecal samples have a big advantage in the low cost and non-invasiveness of the sampling, but the composition of the fecal bacterial population can be extremely different from the mucosal (ileal or colonic) one [6,44,45]. Only the well-detailed case-report by D’Argenio et al. [28] evaluated the intestinal microbiota based on histological samples. However, the importance of the sampling site is minimized by looking at the longitudinal changes induced by the EEN treatment. Both antibiotics and probiotics have been demonstrated to significantly affect the bacterial microbiota community in the GI tract [46,47]. Furthermore, bowel preparation can affect the composition and the diversity of both fecal and luminal microbiota [48]. As previously stated, only six of the considered studies in this review clearly indicated the use of antibiotics as an exclusion criteria, and, in two studies, patients on probiotics were excluded; additionally, bowel preparation was reported only in two studies [23,35]. We should consider these methodological pitfalls as possible limitations when extending the results of this review.

Overall, the more recent and well-documented studies seem to indicate a paradoxical effect of EEN on the intestinal microbiota. In fact, a reduction in microbial diversity and/or richness induced by EEN was found in three recent and well-designed studies [29,30,32] that all together examined 43 CD patients on EEN. The diversity at the end of EEN was found to be significantly reduced in comparison with both the microbial diversity of pre-treatment samples and the microbial diversity of healthy controls. This effect reverses following the resumption of a normal diet [29,30]. The transitory effect of EEN could explain the reason why this dietetic approach has a very high efficacy as an induction treatment and is able to induce mucosal healing but is less effective in maintenance of remission. These results are in accordance with animal studies [49], demonstrating a decrease in the diversity of bacterial species in interleukin-10 (IL 10) deficient mice fed with an elemental diet compared with mice fed with the regular diet. Interestingly, although limited by the paucity of data and the early techniques, in healthy subjects enteral nutrition also seems to induce a reduction of the total intestinal bacterial count [50]. The same effect has been described in pigs fed with EEN compared to those receiving TPN [51]. Possible explanations are the absence of dietary fiber, with consequent reductions in the exogenous carbohydrate available for fermentation, and/or an increase in intestinal transit time, which may independently reduce microbial mass [52].

The effects of the therapeutic formula on specific bacterial strains were very variable and not consistent between studies. This is not surprising considering both the small sample sizes and the variable methods used in different studies and the huge degree of inter-individual variation in microbiota composition normally encountered in different people. More frequently, studies reported a decrease in the abundance of Firmicutes following a course of EEN [25,29,30], thus increasing the difference of the IBD microbiota subjects from the healthy subjects’ bacterial composition. In fact, in patients with IBD, a reduced representation of Firmicutes and a parallel increase in Proteobacteria are generally reported [53,54,55]. It is plausible, however, that this effect simply reflects a general depletion of all the species already present in the gut at the start of the intervention rather than being a specific effect induced by EEN. In a recent review on the therapeutic utility of EEN in CD, Cuiv and their co-authors suggest that the effect of EEN on intestinal bacteria is mainly based on the limitation of growth and metabolic activity rather than the selection of a specific microbiological pattern [56]. The same authors proposed that the bowel rest induced by EEN may induce mucosal healing by limiting the activity of potentially pathogenic microbes and by enhancing repairing mechanisms (autophagy) [56]. The reduction at the end of EEN of *F. prausnitzii*, a butyrate producing bacteria considered to be protective towards CD development [37,38], reported by some studies [25,29,30] is in agreement with this hypothesis. In fact this reduction could simply be the expression of the general effect induced by EEN. Interestingly, relevant changes in abundance of the AIEC, another bacteria described as potentially pathogenic in IBD [57,58,59], were not reported in any of the studies. This lack of consistency in pointing out possible community differences might indicate that efforts should be probably directed towards identifying other potentially more relevant discrepancies; for example, at a metabolic or functional level.

## 6. Conclusions

Currently there is limited data on the microbiological effect induced by EEN in subjects with CD. However, the most recent and well-documented results suggest a paradoxic effect of EEN, consisting in a reduction of bacterial diversity and richness. Long-term, multicenter, and rigorous studies based on NGS technology comparing the effect of different treatments are expected to clarify the relevance of microbiological changes induced by the EEN.

## Figures and Tables

**Table 1:** Characteristics and main outcomes of the 14 studies included in the review.

Author, Year	Type of Study	Groups of Subjects (Number, Characteristics)	Exclusion Criteria (ATBs, Probiotics, Other Drugs)	Biological Sample (Type and Number of Samples)	Type of Formula and Duration of Treatment	Microbiota Analysis	Outcomes
Lionetti P et al., 2005 [23]	Prospective, controlled	nine active Crohn’s Disease (CD) adolescents (nine to 17 years), five controls (10–15 years)	Antibiotics or colon cleansing in the previous week	Fecal samples, multiple samples during the exclusive enteral nutrition (EEN) course and during partial enteral nutrition (total number not indicated)	Polymeric formula enriched with TGF-β2 for eight weeks	Temperature gradient gel electrophoresis (TGGE) analysis of 16S rRNA	TGGE profile varied greatly between subjects and required time to achieve stability of the band profile in each subject during exclusive and partial EN (no statistical analysis available).
Leach ST et al., 2008 [24]	Prospective, controlled	six CD children at diagnosis, mean age 10.2 years (2.5–13.5); seven controls, mean age 5.9 years (2.1–12)	Antibiotics or antiinflammatory agents in the previous four weeks. Severe CD requiring surgery or intensive medical treatment.	Fecal samples collected prior to endoscopy and at one, two, four, six, eight, 16 and 26 weeks after the start of EEN	eight weeks of EEN (formula not specified)	PCR amplification of the bacterial 16S rRNA gene followed by denaturating gel electrophoresis (DGGE)	CD children had a greater degree of change in the bacterial composition during EEN compared to controls on a normal diet (*p* < 0.05). The greatest change was seen in Ruminococcaceae (*p* < 0.001) and the least in the *Bacteroides*-*Prevotella* group (*p* < 0.01).
Jia W et al., 2010 [25]	Prospective, controlled	20 CD, 21 Irritable bowel syndrome (IBS), 14 Ulcerative colitis (UC), and 18 controls	Not indicated	Fecal samples collected before and after two weeks of EEN treatment	two weeks of elemental formula	PCR amplification of *Faecalibacterium prausnitzii* DNA (A2-165 and M21/2 subgroup)	Levels of *F. prausnitzii* A2-165 decreased significantly (*p* = 0.0046) after treatment compared to baseline and to other groups. Levels of *F. prausnitzii* M21/2 decreased without statistical significance (*p* = 0.61).
Shiga H et al., 2012 [26]	Prospective, controlled	33 active CD (median age: 30 years, 15–47), 17 controls	No antibiotic or probiotics during the study period.	Fecal samples at baseline, after 38 days for the EEN group, 35 days for the total parenteral nutrition (TPN) group, six weeks for the controls. In 12 healthy controls, a second fecal sample was collected after six weeks.	eight patients: eight weeks of elemental formula; nine patients on total parenteral nutrition	Terminal restriction fragment length polymorphism analysis of bacterial 16S rRNA to evaluate the whole microbiota. Specific quantitative PCR to determine predominant bacterial groups.	Number of bacterial species was reduced by EEN in CD (*p* = 0.672), the ratios of bifidobacteria and *Bacteroides fragilis* were reduced (*p* = 0.664 and 0.034, respectively), and *Enterococcus* was increased (*p* = 0.788).
Tjellstrom B et al., 2012 [27]	Prospective controlled	18 active CD children, median age 13.5 years (10–17); 12 healthy controls, median age 14.5 years (14–15.5)	Not indicated	Fecal samples (eight patients collected at the start and finish of EEN)	six weeks of polymeric formula	Determination of the fecal pattern of short-chain-fatty acids (SCFAs) using gas-liquid chromatogrphy	Concentration of fecal acetic acid was reduced by EEN (*p* < 0.05), and butyric and valeric acids were increased (*p* = ns). 79% of CD showed response to EEN, showing a fecal pattern of SCFAs similar to healthy children.
D’Argenio et al., 2013 [28]	Case report, controlled	one active CD patient (14 years), one control with gut polyp (15 years)	Not indicated	Ileum samples	eight weeks of polymeric formula	16S rRNA next-generation sequencing	Bacterial diversity was reduced in CD patient at baseline compared to control and increased after EEN (*p* < 0.05). Composition changed after therapy (Bacteroides increased and Proteobacteria decreased) reaching a distribution similar to the healthy control (statistical significance not indicated).
Gerasimidis K et al., 2014 [29]	Prospective, controlled	15 active CD children (median age: 12.7 years), 11 newly diagnosed and four started a second EEN course; 21 healthy controls (median age: 9.9 years)	Antibiotics in the previous 3 months	68 fecal samples from CD subjects (baseline, 15 to 30 days on EEN, at EEN end, and two to four months after EEN). 40 samples from controls (two samples for each)	eight weeks of polymeric formula TGF-β2 enriched	16S rRNA amplification and quantification with real-time quantitative PCR. Measurement of SCFAs by gas cromatography. Measurement of D and L-lactate by enzymatic commercial assay. Measurement of fecal sulfide by a spetrophometric method.	After EEN, the global bacterial diversity abundance decreased (*p* = 0.037) and returned to normal on a free diet (*p* = 0.041). During EEN, concentrations of *F. prausnitzii* (*p* = 0.002) and *Bifidobacterium* genus decreased (*p* = 0.053) and re-increased on a normal diet (*p* = 0.006 for Faecalibacterium, *p* = ns for Bacteroides/Prevotella), but remained lower compared to healthy subjects.
Quince C et al., 2015 [30]	Prospective controlled	23 active CD (age: 6.9–14.7 years) 21 controls (age: 4.6–16.9 years)	Antibiotics in the previous three months	78 fecal samples from CD patients (baseline, during EEN: 16th to 32th and 54th day and 63 days after EEN) 39 fecal samples from controls (collected at least two months apart)	eight weeks of polymeric formula TGF-β2 enriched	Sequencing of 16S rRNA gene performed on the MiSeq platform. Shotgun metagenome sequencing was performed for 69 samples with the Nextera XT Prep Kit and the Illumina dual barcoding Nextera XT Index kit. Shotgun metagenomics reads were used also for assignment to functional models through alignment to Kyoto Encyclopedia of Genes and Genomes (KEGG).	A decrease in species was evident after 15 days of EEN (*p* = 0.037). Diversity returned to baseline when patients were back to a normal diet but remained lower (at any time) compared to controls. At the community level, EEN made the CD microbial even more dissimilar to that of healthy controls. 34 genera significantly were reduced over the EEN course (including *F. prausnitzii*); only *Lactococcus* increased with EEN.
Lewis JD et al., 2015 [31]	Prospective controlled	90 active CD children (age: 10.1–15.5 years): 52 anti-TNF 21 EEN, 16 PEN 26 Healthy controls (age: 7.9–19.9 years, data collected from a previous study)	Probiotics in the previous two weeks, children with an ostomy	366 Fecal samples collected at baseline, one to four and eight weeks into therapy	EEN: 90% of calories from a not specified dietary formula; PEN: 53% of calories from formula	Bacterial DNA sequenced using the Illumina HiSeq method.	Microbiota composition changed within one week of EEN, moving farther from the centroid of healthy controls (overall *p* = 0.05, among responders *p* = 0.02, among non responders *p* = 0.14). Abundance of six genera changed after one week (*p* = 0.05). An opposite pattern was seen in anti-TNF treated patients (microbiota composition became similar to healthy controls in one week) and in PEN treated patients. At the end of the eight weeks both EEN and anti-TNF responders had a microbiota composition similar to healthy controls.
Kaakoush NO et al., 2015 [32]	Prospective, controlled	five newly diagnosed CD children, five healthy controls	Antibiotics or antinflammatory agents in the previous four weeks	39 fecal samples collected at baseline (at diagnosis, prior to bowel cleansing for endscopy) and then at one, two, four, eight, 12, 16, and 26 weeks after diagnosis.	eight to 12 weeks of a polymeric formula	16S rRNA gene and whole-genome high throughout sequencing	The number of OTUs decreased during EEN in responder patients (no statistical analysis indicated).
Schwerd T et al., 2016 [33]	Prospective	15 CD children, 12 newly diagnosed (mean age: 13.5 years, SD: 2.2 years)	Not indicated	24 fecal samples collected from eight CD subjects at baseline, week two, and at cessation of EEN	Polymeric formula TGF-β2 enriched in 14 patients, elemental formula in one patient	High-throughput 16S rRNA gene sequencing	Altered fecal bacteria composition was seen after two weeks of EEN (bacterial profiles clustered from pre-EEN). EEN decreased the abundance of phylum Bacteroidetes (*p* = 0.039) and increased the abundance of Firmicutes (*p* = 0.027).
Guinet-Charpentier C et al., 2016 [34]	Prospective, controlled	34 CD children (median age 14.8 years, range 6.5–21): four children on EEN, eight on PEN, 22 on other treatments	Not indicated	Fecal samples collected at baseline, two weeks, and six weeks after EEN (three patients)	Polymeric formula TGF-β2 enriched	MiSeq sequencing of the 16S rRNA gene	A decrease in genera from the Proteobacteria phylum (particularly *Sutterella*) was observed (*p* < 0.05), whereas *Alistipes* (*p* < 0.05) and *Bifidobacterium* (*p* < 0.1) increased during EEN.
Dunn KA et al., 2016 [35]	Prospective, controlled	10 children with active CD (age 10 to 16 years) on EEN for 12 weeks; five controls (CD relatives) on normal diet	Use of other medications (including antibiotics) was not an exclusion criteria	19 Fecal samples from CD patients collected at baseline (at least 48 h after bowel preparation) and week 12	12 weeks of EEN by NG tube	High-throughput sequencing of the 16S rRNA gene targeting the V6-V8 region performed on the Illumina MiSeq platform	Species diversity (Chao-1 index) decreased among sustained remission (SR) samples, whereas it increased among the non-SR samples over the course. Taxonomic composition changed over the course of EEN treatment (no specific statistic measure is indicated).
Dunn KA et al., 2016 [36]	Prospective, controlled	15 CD patients (aged 10–16 years), five controls (age nine to 14 years, relatives of CD patients)	Use of other medications (including antibiotics) was not an exclusion criteria	33 CD patient samples (15 at baseline and 18 at various times at or after the end of EEN treatment (week 12). Five samples from healthy controls	12 weeks of polymeric formula	Metagenomic data obtained by next-generation sequencing (NGS) (Illumina MiSeq). Sequences were compared to 28 complete microbial genomes annotated with KEGG.	Eight KEGG pathways differed significantly between baseline CD patients and controls (*p* < 0.05). SR patients had greater similarity to controls than NSR patients in all cases.

**Table 2:** Specific bacterial changes induced by exclusive enteral nutrition (EEN).

Increased during EEN	Decreased during EEN
**Firmicutes**	
Relative abundance of Firmicutes (*p* = 0.227) [33]	Levels of A2-165 *Faecalibacterium prausnitzii* (*p* = 0.0046) [25]
	Levels of M21/2 *Faecalibacterium prausnitzii* (*p* = 0.61) [25]
	Concentration (log10 16S Ribosomal RNA Gene Copy Number/g of dry stool) of *Faecalibacterium prausnitzii* (*p* = 0.002) [29]
	Relative abundance of *Faecalibacterium* (*p* = 0.068) [30]
Relative abundance of *Lactococcus* (*p* = 0.017) [30]	Relative abundance of *Dialister* (*p* = 0.04) [30]
Relative abundance of Christensenellaceae (*p* = 0.0237) [33]	Relative abundance of Ruminococcacae (*p* = 0.04) [30]
	Relative abundance of *Subdoligranulum* (*p* = 0.023) [30]
**Bacteroidetes**	
Concentration of *Bacteroides* (*n* = 1, *p* not reported) [28]	Concentration (log 10 cells per g of faeces) of *Bacteroides fragilis* (*p* = 0.034) [26]
Abundance of *Alistipes* (*p* < 0.05) [34]	Concentration of *Bacteroides*/*Prevotella* (*p* = 0.053) [29] Concentration of *Prevotella* (*p* = 0.27) [30]
	Relative abundance of Bacteroidetes (*p* = 0.039) [33]
**Proteobacteria**	
	Concentration of Proteobacteria (*n* = 1, *p* not reported) [28]
	Concentration of *Sutterella* (*p* < 0.05) [34]
**Actinobacteria**	
Abundance of *Bifidobacterium* (*p* < 0.1) [34]	Abundance of Bifidobacteriaceae genus (*p* = 0.005) [30]
	Concentration of *Bifidobacteria* (*p* = 0.003) [29]
